# Electrochemical Tracking of Macrophage Migration Inhibitory Factor: A Leap Toward Precision Colorectal Cancer Diagnosis and Prognosis

**DOI:** 10.3390/bios15110739

**Published:** 2025-11-04

**Authors:** Eloy Povedano, Antonino-Biagio Carbonaro, Verónica Serafín, María Gamella, Alessandro Giuffrida, Ana Montero-Calle, José Manuel Pingarrón, Rodrigo Barderas, Susana Campuzano

**Affiliations:** 1 Department of Analytical Chemistry, Faculty of Chemical Sciences, Complutense University of Madrid, 28040 Madrid, Spain; elpove01@ucm.es (E.P.); antonino.carbonaro@phd.unict.it (A.-B.C.); veronicaserafin@quim.ucm.es (V.S.); mariagam@quim.ucm.es (M.G.); 2 Department of Chemical Sciences, University of Catania, 95100 Catania, Italy; alessandro.giuffrida@unict.it; 3 Chronic Disease Programme, UFIEC, Institute of Health Carlos III, Majadahonda, 28220 Madrid, Spain; ana.monteroc@isciii.es (A.M.-C.); r.barderasm@isciii.es (R.B.); 4 CIBER of Frailty and Healthy Aging (CIBERFES), Institute of Health Carlos III, 28046 Madrid, Spain

**Keywords:** macrophage migration inhibitory factor MIF, electrochemical biotechnology, diagnosis, prognosis, staging, colorectal cancer

## Abstract

Colorectal cancer (CRC) remains a significant global health burden, mainly due to late diagnosis and chemotherapy resistance. Macrophage migration inhibitory factor (MIF), a proinflammatory cytokine associated with tumor progression, has emerged as a promising biomarker in CRC. However, its clinical utility is limited by the lack of rapid and accessible detection methods. In this study, we report an electrochemical immunotechnology for the sensitive and selective quantification of MIF protein in CRC tissue samples. By combining magnetic microparticles (MMPs), antibody-based recognition, horseradish peroxidase (HRP) labeling, and amperometric transduction at disposable screen-printed carbon electrodes (SPCEs), the developed methodology displayed a linear dynamic range from 0.24 to 20 ng mL^−1^, enabling quantification across clinically relevant MIF levels, and achieving a low limit of detection (0.07 ng mL^−1^). In addition, the developed method is the only one reported for MIF assembled on MMPs and addresses its determination in a relevant oncological scenario (paired non-tumoral (NT) and tumoral (T) tissues from individuals diagnosed with CRC at different stages of the disease). The analysis, requiring only 100 ng of tissue extract, allowed efficient discrimination between NT and T paired tissues, and successfully differentiated between healthy, early (I–II) and advanced (III–IV) CRC stages, achieving these results in just 105 min.

## 1. Introduction

Colorectal cancer (CRC) is ranked among the three most frequently diagnosed malignancies, and the second leading cause of cancer-related mortality worldwide [1,2]. Indeed, it is estimated that it is responsible for nearly half a million deaths per year [3,4]. A significant risk factor in CRC is the development of liver metastasis, which occurs in approximately 50% of patients, and represents a major treatment obstacle as well as a leading cause of death [5,6]. In fact, the five-year survival rate is highly dependent on tumor stage at diagnosis, with early-stage tumors showing a favorable prognosis of approximately 90%, which declines to 12% or less in advanced metastatic stages [2,3]. It is also important to highlight that early CRC is usually asymptomatic and clinical symptoms often appear only at stage IV, when metastatic spread has already occurred, contributing to late diagnosis and reduced survival chances [3,7,8].

Despite significant advances in the diagnosis and treatment of CRC, its prognosis remains poor due to its high potential for recurrence and metastasis [8]. Although chemotherapy is the mainstay of CRC treatment [9], resistance to therapy, combined with the usually late disease diagnosis, constitutes the leading cause of therapeutic failure, disease progression, and discouraging prognoses [10,11,12]. In this obscure scenario, early detection of CRC is crucial for effective therapeutic management and improved patient prognosis and survival. Understanding the molecular mechanisms underlying CRC invasiveness, metastasis, and chemoresistance is key to increasing therapeutic efficacy and improving patient prognosis [8]. Consequently, the discovery and analysis of new biomarkers have become a priority for global health, as it not only enables early, minimally invasive, and more accurate detection of CRC, but also allows for more effective treatments to be applied and their outcomes to be predicted and monitored.

Macrophage migration inhibitory factor (MIF) stands out as one of these promising biomarkers. Since its activity was first reported in the mid-1960s, MIF has evolved from a simple cytokine modulating macrophage motility to a multifunctional regulator involved in a wide range of cellular and biological processes [13,14]. Among its functions, MIF is involved in numerous regulating biological processes such as cell proliferation, apoptosis, migration, and invasion by interacting with its abundant receptors and activating multiple signaling pathways. These interactions allow MIF to influence inflammation, immune response, and tumor progression [15]. In this context, it has been identified as a pro-inflammatory cytokine in several immune diseases, including rheumatoid arthritis and lupus erythematous [16]. Recent studies have shown that MIF also plays a significant role in the occurrence, progression, and resistance of several solid tumors [8,17] including head and neck [18], lung, breast, prostate, cervical, hepatocellular, gastric, esophageal, glioblastoma, renal, pancreatic, neuroblastoma, melanoma, ovarian cancer [19], and CRC [5,8,20,21].

Aberrant overexpression of MIF in CRC has been closely linked with tumor aggressiveness, invasiveness, metastasis, poor prognosis, and resistance to chemotherapy [8,17,19,22]. Indeed, it has been reported that MIF is upregulated in serum and tissues of patients with CRC compared to healthy individuals [5,23]. Moreover, MIF is more specific and more sensitive than the carcinoembryonic antigen (CEA) in detecting CRC [24,25]. MIF also contributes to chemotherapy resistance in CRC, and although the specific mechanisms remain unclear, it is emerging as a key player in this process, continuing to hinder effective clinical outcomes. Thus, there is an urgent need to develop methods for accurately quantifying MIF levels to support diagnosis, prognosis and plan personalized treatments, predict therapeutic response, and guide new pharmacological strategies to overcome this type of treatment-resistant cancer.

Each of these techniques offers important advantages, such as high sensitivity, specificity, and quantitative accuracy. However, they also present certain limitations, including variability between assays, technical complexity, high cost, and dependence on sample quality. Therefore, the careful selection and combination of complementary methods are crucial to ensure reliable and reproducible biomarker analysis. Recent advances in multiplex immunoassays and mass spectrometry have further enhanced the sensitivity and specificity of biomarker profiling, offering new opportunities for translational research and clinical applications.

Commonly employed methodologies for MIF detection and quantification include enzyme-linked immunosorbent assays (ELISAs) [26], Western blotting [27], quantitative real time PCR [28], and mass spectrometry [29]. Even though these techniques provide reliable, sensitive, and selective results, they also suffer from certain limitations, including technical complexity, high cost, long processing times, and specialized laboratory infrastructure and personnel. These limitations hinder their broad application, particularly in resource constrained environments. Therefore, it is necessary to explore the development of complementary or improved methodologies able to overcome such limitations to provide a more comprehensive understanding of this biomarker dynamics.

In this context, electrochemical immunotechnologies have emerged as promising complementary tools to redefine biomarker detection. Unlike other methods, they combine the high specificity of antigen–antibody interactions with the sensitivity of electrochemical transduction. Their fast response, sensitivity, and low cost, coupled with their portability, miniaturization, and multiplexing potential, position them as particularly valuable tools for the monitoring of biomarkers at the point-of-care [30]. Among these immunotechnologies, those involving magnetic microparticles (MMPs) as scaffolds for assay assembly have aroused significant attention due to the inherent advantages conferred by their magnetic properties. Their use facilitates rapid and efficient separation, concentration and purification of target analytes, thereby improving sensitivity, shortening determination time and minimizing matrix effects, which is critical while dealing with complex intricate biological samples [31,32]. MMPs electrochemical systems exhibit outstanding analytical performance and versatility, while offering scalability and straightforward adaptation to decentralized diagnostic workflows. To our knowledge, there are only two electrochemical immunosensors reported in the literature for the determination of MIF, and none of them involve the use of MMPs. These immunosensors were applied in clinically relevant scenarios (rheumatoid arthritis [33] and allergic rhinitis [34]) different than cancer.

Accordingly, we describe in this work a rapid and straightforward electrochemical sandwich immunoplatform for the determination of MIF through the combination of MMPs, enzymatic labeling with horseradish peroxidase (HRP), and amperometric transduction at screen-printed carbon electrodes (SPCEs). The developed approach not only demonstrated excellent analytical and operational performance but also showed a high capacity to determine MIF levels in tissue samples from CRC patients at different stages.

## 2. Materials and Methods

### 2.1. Apparatus, Instruments and Electrodes

A potentiostat (model 812B, CH Instruments, Austin, TX, USA) controlled by the CHI812B software was used to conduct all amperometric readings at room temperature. Screen-printed carbon electrodes with a single working position (SPCE, DRP-110, 4 mm diameter) were used as electrochemical transducers to carry out the amperometric determinations, and a specific cable connector (DRP-CAC) was used to connect SPCE to the potentiostat. Both were purchased from Metrohm-DropSens S.L. (Asturias, Spain).

A homemade polymethyl methacrylate (PMMA) block with a neodymium magnet (AIMAN GZ) embedded inside, placed exactly under the working electrode (WE) when the SPCE was placed on it, was used to achieve stable and reproducible fixation of the magnetic immunoconjugates.

The following apparatus were also used: a tube mixer AGT-9 (Bunsen, Madrid, Spain) for the homogenization of the solutions, a steam sterilizer (Raypa, Terrassa, Barcelona, Spain), a biological safety cabinet (Telstar Biostar, Terrassa, Barcelona, Spain) for handling of biological samples, an incubator shaker (Optic Ivymen^®^ System, Comecta S.A., Barcelona, Spain), a precision Crison Basic 20+ pH-meter (Orion Star A214 model, Thermo Fisher Scientific, Alcobendas, Madrid), and a magnetic concentrator (DynaMag™-2, 123.21D, Invitrogen Dynal AS, Oslo, Norway).

### 2.2. Reagents and Solutions

All the reagents used were of the highest available analytical grade. MMPs modified with carboxylic acid groups (COOH-MMPs, ϕ = 2.7 μm, 10 mg mL^−1^, Dynabeads M-270 Carboxylic acid, Cat. No. 14305D) were purchased from Life Technologies S.A. (Alcobendas, Madrid).

Mouse anti-Human MIF Capture Antibody (CAb), biotinylated goat anti-Human MIF Detection Antibody (bDAb), and recombinant Human MIF Standard were components of the Human MIF kit (Cat. No. DY289) purchased from R&D Systems (Minneapolis, MN, USA).

A blocker casein solution (consisting of 1% *w*/*v* purified casein ready-to-use PBS solution, BB) and a high-sensitivity streptavidin-horseradish peroxidase conjugate (Strep-HRP) were purchased from Thermo-Scientific and Roche (Basel, Switzerland), respectively.

Other reagents such as *N*-(3-dimethylaminopropyl)-*N*′-ethylcarbodiimide (EDC), ethanolamine (ET), hydroquinone (HQ), and hydrogen peroxide (H_2_O_2_, 30% *w*/*v*) were acquired from Sigma-Aldrich (Saint Louis, MO, USA); NaCl, KCl, NaH_2_PO_4_, Na_2_HPO_4_, and Tris-HCl were purchased from Scharlab (Barcelona, Spain); 2-(*N*-morpholino)ethanesulfonic acid (MES) was purchased from Gerbu (Heidelberg, Germany); and *N*-hydroxysulfosuccinimide (sulfo-NHS) was provided from Fluorochem (Hadfield, UK).

Human IgG (hIgG, Cat. No. I2511), human albumin (HSA, Cat. No. A1653), and human hemoglobin (Hb, Cat. No. H7379), evaluated as potential interferents, were acquired from Sigma-Aldrich.

All buffer solutions were prepared in Milli-Q water (18 MΩ cm at 25 °C): PBS consisting of 10 mM phosphate buffer solution (PB) containing 137 mM NaCl and 2.7 mM KCl (pH 7.5), Tris-HCl (10 mM, pH 7.2), MES (25 mM, pH 5), PB (50 mM, pH 6) and PB (100 mM, pH 8).

### 2.3. Preparation of Magnetic Immunoconjugates

The COOH-MMPs modification for the immunoplatform assembly was conducted in 1.5 mL microcentrifuge tubes using an incubator shaker (25 °C, 950 rpm) for successive incubations with 25 µL of the appropriate bioreagent. After each incubation step, MMPs were washed twice with 50 µL of the corresponding buffer solution. All incubation and washing steps were assisted by a magnetic concentrator that allows efficient magnetic separation of the MMPs (3 min) to remove the supernatant easily.

Independent microcentrifuge tubes were used for each determination. Briefly, 3.0 µL-aliquots of COOH-MMPs were dispensed in each tube and washed twice (10 min each) with MES buffer. Next, the MMPs’ carboxyl groups activation was performed via a 35 min incubation with a freshly prepared mixture solution of EDC/NHSS (50 mg mL^−1^ each), followed by the corresponding washings with MES buffer. The covalent binding of the biorecognition element was conducted by incubating the activated COOH-MMPs in the CAb solution (25 μg mL^−1^, prepared in MES buffer) for 30 min. Once washed with MES buffer, a blocking step of the free residual activated COOH groups was carried out with ethanolamine (ET) (1.0 M, dissolved in PB 100 mM, pH 8) for 60 min. After washing with Tris-HCl buffer and twice with BB solution, the blocked CAb-MBs were stored at 4 °C in filtered PBS until use.

The sandwich immunoassay was assembled on the blocked CAb-MMPs by sequentially incubating with the synthetic MIF standard (or the analyzed sample, both prepared in PBS) for 60 min, the bDAb (1.0 μg mL^−1^, prepared in BB) for 30 min and Strep-HRP conjugate (1000-fold diluted in BB) for 30 min, intercalating between each incubation washings with BB solution. The functionalized MMPs were re-suspended in 50 µL of PB (50 mM, pH 6) to conduct amperometric measurements.

### 2.4. Amperometric Measurements

For each measurement, a new SPCE was placed in the homemade PMMA housing, and the re-suspended functionalized MMPs were captured on the WE by drop casting.

The assembly PMMA support/SPCE-MMPs was connected to the potentiostat via the specific connector cable and immersed into an electrochemical cell containing 10 mL of a freshly prepared 1 mM HQ solution in PB (50 mM, pH 6). At room temperature and under continuous mechanical stirring, a constant potential difference of −0.2 V relative to the Ag pseudo-reference electrode was applied. As soon as the background current stabilized, 50 μL of an H_2_O_2_ solution (0.1 M) were added to the electrochemical cell, and the variation of the cathodic current corresponding to the HQ-mediated enzymatic reduction of H_2_O_2_ was recorded until the steady state was reached, which usually occurred after approximately 60 s.

The amperometric responses provided in the manuscript correspond to the difference between the background and steady-state currents, and, unless otherwise specified, these values correspond to the average of three independent replicates. Error bars were calculated as the standard deviation (SD) of the replicates (confidence intervals calculated for α = 0.05).

### 2.5. Protein Extracts from Paired Non-Tumoral and Tumoral CRC Tissues and Statistical Analysis

Optimal cutting temperature (OCT)-embedded frozen tumoral (T) tissue samples from stages I–IV CRC patients, along with paired non-tumoral adjacent tissue (NT), verified by a pathologist, were obtained from the biobank of San Carlos Clinical Hospital, following approval by the Institutional Ethics Committee (CEI PI 13_2020-v2). All participants provided written informed consent before inclusion in the study. Tissue extracts were prepared according to the protocol described previously [35] and total protein concentration was quantified by tryptophan quantification method [36]. Protein extracts were stored at −80 °C and handled in compliance with all applicable ethical guidelines, standards, and regulations for sample processing and experimental procedures.

All tissue extracts were analyzed in triplicate, using 100 ng per determination and employing the standard additions method. Results are presented as mean ± standard error of the mean (SEM). Data plotting, mean calculations, and SEM values were obtained using Microsoft Excel 2019. Receiver operating characteristic (ROC) curves were generated in R (version 3.6.2) using the ‘pROC’ package to evaluate diagnostic performance. The area under the curve (AUC), sensitivity, specificity, and 95% confident interval (95% CI) were obtained with the ‘pROC’ package. Mann–Whitney U tests were also performed in R (version 3.6.2), and *p*-values ≤ 0.05 were considered statistically significant.

## 3. Results and Discussion

This work reports on the development of an amperometric immunoplatform for the determination of MIF involving the use of MMPs and SPCEs. To the best of our knowledge, it is the first immunoplatform of these characteristics reported to date in the literature.

**Figure 1:**
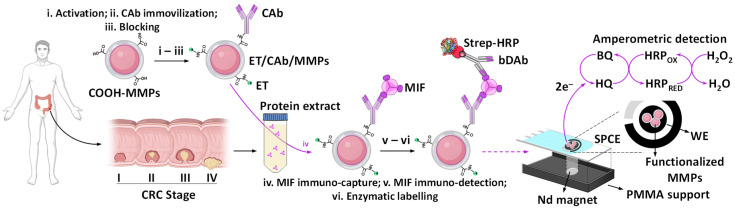
Graphical display of the preparation steps involved in the development of the sandwich-type MMPs-assisted immunoplatform for the determination of MIF, as well as the amperometric transduction process. Created in part with BioRender. Campuzano Ruiz, S. (2025) https://BioRender.com/0q5y3zf.

As shown in Figure 1, the strategy implies a couple of specific capture (CAb) and biotinylated detection (bDAb) antibodies for MIF protein sandwiching on the COOH-MMPs surface, and the use of a high-sensitivity commercial streptavidin-peroxidase conjugate (Strep-HRP) for enzymatic labeling. The resulting immunoconjugates are captured on the working electrode surface (WE) of SPCEs by simply magnetic attraction employing a PMMA home-made support provided with a neodymium magnet positioned just below the WE location. The amperometric transduction is performed in stirred solutions at −0.2 V (vs. Ag pseudo-reference electrode) in the presence of the HQ/HRP/H_2_O_2_ system. According to the designed sandwich immunoassay format, the recorded cathodic current variations, due to the HQ-mediated enzymatic H_2_O_2_ reduction, are directly proportional to the MIF protein concentrations.

### 3.1. Reliability and Fine-Tuning of the Methodology

**Figure 2:**
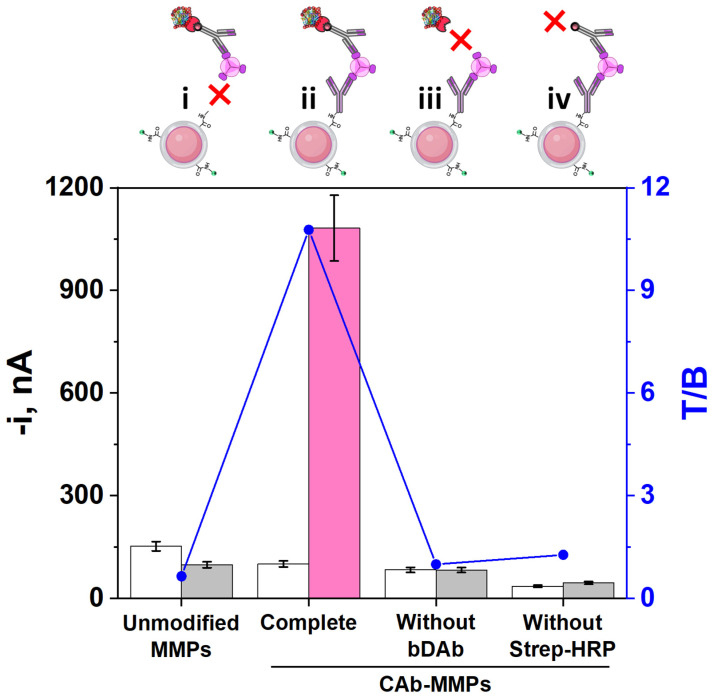
Comparison of the amperometric responses obtained with the developed immunoplatform in the absence (0.0 ng mL^−1^, white bars, B) and in the presence (5.0 ng mL^−1^, fuchsia/grey bars, T) of MIF standard as well as the resulting target to blank measurement ratio (T/B, blue line), with the responses recorded with immunoplatforms employing unmodified MMPs (without immobilized CAb) and CAb-MMPs in the absence of bDAb or Strep-HRP. Created in part with BioRender. Campuzano Ruiz, S. (2025) https://BioRender.com/wy9g8bf.

A thorough assessment of the appropriate performance of the proposed methodology was carried out to ensure its reliability. For this purpose, as shown in Figure 2, a set of control experiments were carried out by comparing the amperometric responses obtained with the developed sandwich-type immunoplatform (Figure 2, configuration ii) and with immunoplatforms constructed without key bioreagents such as CAb, bDAb and Strep-HRP (Figure 2, configurations i, iii, and iv, respectively). As expected, the only significant differences between the measurements recorded in the absence (0.0 ng mL^−1^, white bars, B) and in the presence (5.0 ng mL^−1^, fuchsia/grey bars, T) of MIF were observed when all the components of the sandwich immunoassay were involved (‘Complete’ bars in Figure 2). Also as expected, the rest of the immunoplatforms could not provide any discrimination between both measurements. These results confirmed, besides the rationale of the strategy, the minimal non-specific adsorptions of MIF, bDAb, and Strep-HRP on the surface of unmodified or CAb-MMPs, and the reliability of the amperometric responses obtained for the determination of the target protein.

After confirming the correct rationale of the methodology, the different key experimental variables involved in its preparation and operation were sequentially optimized to obtain the best performance in terms of simplicity and reduced assay time, as well as to reach the required sensitivity to detect the low MIF protein levels found in real scenarios.

**Figure 3:**
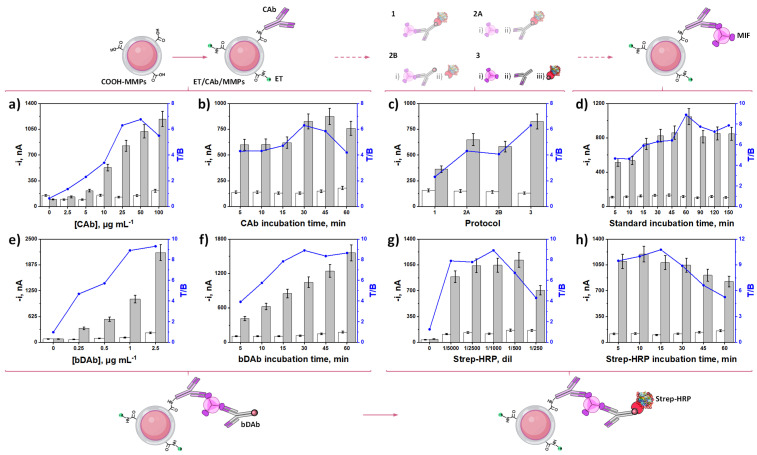
Dependence of the amperometric responses provided by the developed immunoplatform in the absence (0.0 ng mL^−1^, white bars, B) and in the presence (5.0 ng mL^−1^, grey bars, T) of MIF standard as well as the resulting target to blank ratio (T/B, blue line) with CAb concentration (**a**) and incubation time (**b**), steps involved in the assay protocol (**c**), MIF incubation time (**d**), bDAb concentration (**e**) and incubation time (**f**), Strep-HRP conjugate dilution (**g**) and incubation time (**h**). Created in part with BioRender. Campuzano Ruiz, S. (2025) https://BioRender.com/hrdb8vw.

The results obtained are displayed in Figure 3. For each tested variable, the amperometric responses measured at −0.2 V (vs. Ag pseudo-reference electrode) in the absence (white bars, B) and in the presence (grey bars, T) of 5 ng mL^−1^ of MIF standard, were compared. In all cases, the highest ratio between these two currents (T/B ratio, blue line) was selected as the criterion for selection of the optimal values.

Other variables involved in the preparation of the immunoplatform, such as the volume of the COOH-MMPs commercial suspension used, or the concentrations and incubation times of the EDC/NHSS and ET reagents involved in the activation and blocking steps [37,38], as well as variables regarding the amperometric measurements, such as the detection potential, pH, and composition of the supporting electrolyte, and the concentrations of the enzymatic substrate and redox mediator (H_2_O_2_ and HQ, respectively) [39,40,41,42] were optimized in previous works and adopted in the present study.

The execution order of the different optimizations was carried out according to the sequential steps of the immunoplatform assembly thus, starting with the concentration and immobilization time of the biorecognition element, CAb, on the surface of the MMPs (Figure 3a,b). As can be observed, there is a noticeable increment in the specific amperometric responses obtained with the immunoplatform over the whole concentration range tested (0–100 µg mL^−1^). However, the specific signal growth above 25 µg mL^−1^ was smaller, most likely because MIF biorecognition is hindered by steric hindrance when large CAb loadings were immobilized. In addition, increased non-specific adsorptions were observed also above 25 µg mL^−1^ CAb leading to a decrease in the T/B ratio [37]. Similar conclusions can be drawn from the effect of the CAb incubation time (Figure 3b). Incubation times exceeding 30 min resulted in a reduction of the T/B ratios [37]. Accordingly, 25 µg mL^−1^ CAb and 30 min for incubation were selected for the following experiments. No significant fluctuations were observed in the absence of MIF protein (non-specific signals, white bars), demonstrating both the lack of cross-reactivity between the pair of antibodies and non-specific adsorptions on the MMPs surface.

Next, to ensure the use of the most straightforward and sensitive immunoplatform preparation procedure, different protocols all involving 30 min incubation steps from the blocked CAb-MMPs were evaluated (Table 1).

As shown in Figure 3c, larger specific amperometric responses and T/B ratios were obtained when applying the 3-step protocol, conversely to that observed when combining the incubation steps. In the case of protocol 2A, the lower discrimination obtained can be attributed to the multivalency of streptavidin, which has four biotin-binding sites, and can generate cross-reactivity with multiple bDAbs, causing a single Strep-HRP molecule to label several dDAbs molecules when both bioreagents are together in solution [43]. The poor performance obtained while applying protocol 2B can be ascribed to worse CAb recognition towards the MIF protein, probably due to steric hindrance when MIF and bDAb are in the same solution. Finally, the protocol involving a single incubation step shows the adverse synergistic effect produced by the combination of the two previously mentioned effects.

The effect of antigen incubation time on CAb-MMPs is shown in Figure 3d, and it was evaluated over the 5–150 min range. As observed, a 60-min incubation time was enough to achieve the best T/B ratio and, consequently, it was selected for further work.

Finally, the variables implied in the enzymatic labeling stage, including bDAb and Strep-HRP concentrations and the corresponding incubation times, were optimized. Regarding bDAb concentration (Figure 3e), there was a sharp improvement in the specific amperometric responses as well as in the T/B ratio as the bDAb concentration increased. However, considering the smaller increase in the T/B ratio for bDAb loadings larger than 1 µg mL^−1^, this value was selected to strike the balance between sensitivity and cost per determination.

T/B discrimination enhanced with Strep-HRP amount up to a 1/1000 dilution. Smaller dilutions provoked a decrease in the ratio primarily due to smaller specific signals, likely caused by agglutination phenomena that become more prominent at higher Strep-HRP concentrations (Figure 3g). It is important to note that no significant variations of the nonspecific responses were observed when increasing concentration of bDAb or decreasing dilution of the Strep-HRP conjugate, which rules out their significant nonspecific adsorption on both MMPs and CAb surfaces.

Figure 3f,h show the influence of the incubation times of bDAb and Strep-HRP in the 5–60 min range. Larger T/B discrimination was achieved for incubation times of 30 and 15 min, respectively, which were selected for further work. Table 2 summarizes the ranges studied and the values chosen for each of the experimental variables evaluated.

### 3.2. Analytical and Operational Performance

**Figure 4:**
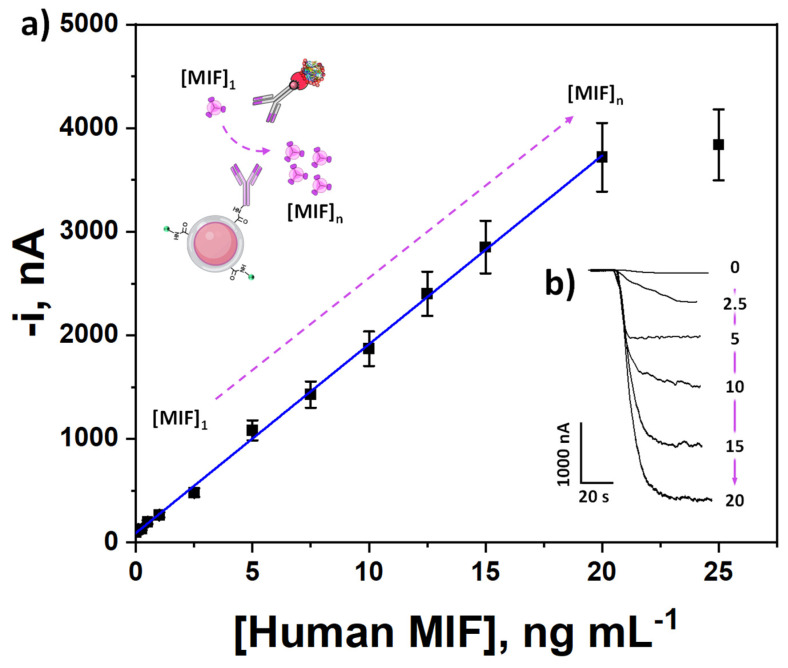
Calibration plot constructed with the developed immunoplatform (**a**) and real amperometric traces recorded for MIF protein determination (**b**). Created in part with BioRender. Campuzano Ruiz, S. (2025) https://BioRender.com/fugg71e.

After optimizing the experimental conditions for the preparation of the immunoplatform, its analytical and operational characteristics were evaluated for the determination of human MIF. The obtained calibration plot is displayed in Figure 4. It exhibited a linear dependence between the amperometric responses and the MIF protein concentration over the 0.24 to 20 ng mL^−1^ range (R^2^ = 0.999), fitting the equation i_c_ (nA) = (182 ± 4) [MIF] (nA mL ng^−1^) + (92 ± 36) (nA). According to the 3 × s_b_/m and 10 × s_b_/m criteria (s_b_: standard deviation of 10 amperometric measurements in the absence of MIF standard and m: slope of the calibration plot), a limit of detection (LOD) of 0.07 ng mL^−1^, and a limit of quantification (LOQ) of 0.24 ng mL^−1^ were calculated, which are below the cutoff value of MIF levels in serum proposed for gastric cancer (3.23 ng mL^−1^) [44], and CRC (34.8 ng mL^−1^) [24].

Table 3 compares the main characteristics of the proposed biotechnology with other methods reported for the determination of MIF.

It is important to note that only two electrochemical immunosensors for the determination of MIF appear in the literature [33,34]. Li et al. developed a label-free electrochemical immunosensor using gold electrodes modified with gold nanoparticles, titanium dioxide nanoparticles, and thionine, onto which IgM monoclonal antibodies were immobilized. The bioplatform achieved a LOD of 0.02 ng mL^−1^ and was applied for quantifying MIF in serum samples of rheumatoid arthritis patients. Although this biosensor exhibited a similar sensitivity to that achieved with the developed immunoplatform, it required remarkably longer preparation time and more efforts as it involved the synthesis of a complex hybrid nanomaterial as well as the in-house production and purification of capture antibodies.

On the other hand, Ma et al. reported a method to assess allergic rhinitis integrating biosensors and intelligent algorithms. High electron mobility transistor immunosensors were fabricated to target MIF, reaching an LOD of 0.018 ng mL^−1^. Although this method achieved a lower LOD than that of our developed immunoplatform (0.07 ng mL^−1^), it did require complex algorithms to discriminate between samples.

Additionally, the determination of MIF was performed using Surface-Enhanced Raman Spectroscopy (SERS). A poly-dopamine chip-MIF-SERS probe involving a sandwich assay with labeled MIF antibody-Au (shell)-Ag (core) nanoparticles as SERS reporters was developed. An LOD value of 0.09 ng mL^−1^ was claimed, and the method was successfully applied to the analysis of Platelet-rich plasma samples [45]. Li et al. reported a polydopamine bi-functionalized SERS immunoassay to detect MIF in exosomes of serum samples of pancreatic cancer patients [46]. These methods exhibited good analytical performance, although SERS instrumentation is generally more costly and technically demanding than electrochemical setups.

On the other hand, the LOD achieved with the developed immunoplatform (0.07 ng mL^−1^) was comparable with those claimed with commercially available ELISA methods with LODs ranging between 0.006 and 0.068 ng mL^−1^ [47,48,49,50,51]. Nevertheless, the developed immunoplatform exhibits remarkable advantages in terms of assay time (1 h 45 min counting from blocked CAb-MMPs vs. 3 h 50 min [50] or 4 h 45 min [51]) (starting from the CAb-coated ELISA plates). Moreover, when compared with other methodologies such as Western blotting, quantitative PCR, or mass spectrometry, the developed approach offers faster analysis, simplified sample handling, and does not require specialized instrumentation or extensive purification steps. Notably, in terms of application, electrochemical immunoplatforms offer greater operational simplicity, lower consumption of reagents and samples, and allow the use of simpler, more affordable, miniaturized and portable instruments, suitable for point-of-need applications. These features make the developed immunotool especially attractive for routine clinical diagnostics and resource-limited settings.

The reproducibility, including both the immunoplatform preparation and amperometric transduction protocols, was evaluated by calculating the relative standard deviation (RSD) of the amperometric signals obtained for 5 ng mL^−1^ of MIF protein with immunoplatforms prepared the same day or in different days. The acceptable RSD values obtained of 8.9% (n = 10) and 7% (n = 6), respectively, indicate consistent performance over time.

In addition, the storage stability of ET blocked CAb-MMPs was tested by preparing and storing them in filtered PBS at 4 °C. Each control day, the amperometric responses obtained with immunoplatforms prepared from the stored CAb-MMPs in the absence and in the presence of 5 ng mL^−1^ of MIF protein were measured. No significant differences in the T/B ratio were observed, compared to that obtained in the preparation batch day (day 0), for at least 38 days (longer times were not studied). These results demonstrate that it is feasible to prepare and store batches of blocked CAb-MMP under the conditions specified above until their use to construct the immunoplatform, so that the determination can be completed in a short time (1 h 45 min).

Interestingly, the time required to carry out the determination can be shortened even further by reducing the bDAb and Strep–HRP incubation times from 30 to 15 and 15 to 5 min, respectively, due to that, under these conditions, the T/B ratio decreased only a 12%. In addition, the simplification of the assay protocol by involving two steps instead of three, would reduce only 21% the specific signal.

### 3.3. Selectivity

The selectivity of the method was evaluated by comparing the amperometric responses obtained for 0.0 (white bars, B) and 5.0 ng mL^−1^ of MIF standard (fuchsia/grey bars, T), in the presence of potential interferents such as human IgG (hIgG), human serum albumin (HSA) or hemoglobin (Hb), which are commonly found in biological samples. In addition, the supplier company (DuoSet ELISATM, R&D Systems) certified the absence of cross-reactivity or interference for the recombinant human IFN-γ and IL-4, or mouse MIF proteins, all of which were assayed at 50 ng mL^−1^. Therefore, the interference of these compounds was not evaluated in this work.

**Figure 5:**
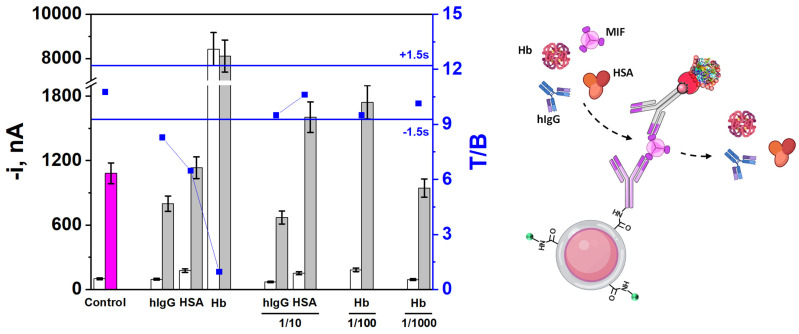
Amperometric responses provided by the developed immunoplatform for 0.0 ng mL^−1^ (white bars, B) or 5.0 ng mL^−1^ (fuchsia/grey bars, T) of MIF standard, prepared in the absence (“Control” bars) or in the presence of the potential interferents hIgG (0.1 mg mL^−1^), HSA (5.0 mg mL^−1^), and Hb (0.005 mg mL^−1^) and upon 10- to 1000-fold dilution. T/B ratio values displayed in blue dots and control limits (blue lines) were set as ± 1.5s of the mean value of three immunoplatforms. Created in part with BioRender. Campuzano Ruiz, S. (2025) https://BioRender.com/69hqt01.

As shown in Figure 5, the three proteins caused significant non-specific interactions—an issue widely reported in the literature—when tested at significant concentrations, compromising the detection of the target protein MIF.

The interference generated by hIgG can be attributed to either its non-specific adsorption on the MMPs surface, or the presence of human anti-mouse antibodies (HAMAs) in serum and the commercial hIgG isolated from it, which can interact with the heavy chains of mouse-expressed immunoglobulins, as the CAb used in this assay [52,53,54,55,56]. HAMAs are heterophilic antibodies naturally occurring in human serum that may compromise immunoassay accuracy by either cross-linking or obstructing assay antibodies, resulting in imprecise readings. While HAMAs production is commonly associated with prior exposure to murine monoclonal antibodies, several studies have reported their presence in approximately 11.7% of serum samples obtained during routine diagnostics [53]. Notably, their elevated prevalence has been observed in CRC patients even in the absence of prior monoclonal antibody treatment [57]. As evidenced by the observed T/B ratio, a simple sample dilution minimizes this interference, making feasible the determination of the MIF biotarget.

On the other hand, a substantial interference was observed in the amperometric responses obtained both in the absence and presence of MIF when incubated jointly with Hb or HSA. This interference was attributable to Hb pseudo-peroxidase activity—widely reported to generate false-positive results—or to the hIgG presence in low-purity HSA batches, respectively [57,58,59], and they have been already observed in other sandwich-type immunosensors implemented on MMPs [56,60].

However, as shown in Figure 3, no significant difference in the T/B ratio was apparent upon 10 times (HSA) or 100 times (Hb) dilution. It is also important to highlight that the presence and concentration of the three evaluated interferents are closely dependent on the type of sample analyzed, being relevant in liquid biopsy samples but not in solid biopsy specimens.

### 3.4. Analysis of Tissues of CRC Patients

The applicability of the developed immunoplatform for the determination of MIF protein was checked by analyzing 100 ng of paired tumoral (T) and non-tumoral (NT) tissue extracts from patients diagnosed with CRC at different stages (I–IV). To test potential matrix effects, a statistical comparison was performed between the slopes of the calibration plots constructed with MIF standards in buffer solutions and those obtained using representative T and NT tissue extracts. As shown in Table 4, the results confirmed the absence of matrix effect (t_exp_ < t_tab_) in both T and NT tissue needle-biopsies when using 100 ng or less of tumor extract. Nevertheless, the standard additions method was chosen to perform the determination. This decision was driven by the restricted sample volume and the inherently low MIF concentration in the 100 ng of the analyzed extract, which resulted in amperometric responses located at the lower limit of the immunoplatform’s linear dynamic range depicted in Figure 4.

**Figure 6:**
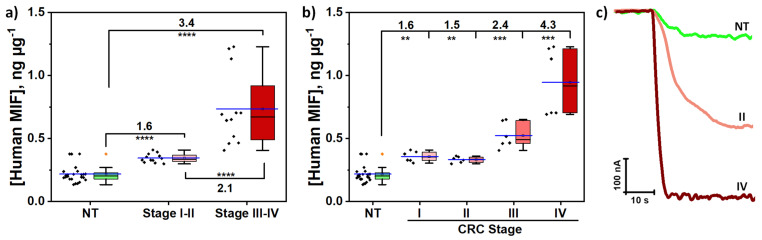
(**a**,**b**) Results obtained with the developed immunoplatform for the determination of MIF protein in 0.1 µg of tissue extracts from paired healthy (NT) and CRC tissues of patients diagnosed at different stages of the disease. The numbers above and the asterisks indicate, respectively, the T/NT ratio and the statistical *p*-value (the higher the number of asterisks, the larger significant differences). (**a**) NT vs. CRC I–II (*p*-value = 0.0000989), NT vs. CRC III–IV (*p*-value = 0.00000146), and CRC I–II vs. CRC III–IV (*p*-value = 0.000002). (**b**) NT vs. CRC stage I (*p*-value = 0.00130324), H vs. CRC stage II (*p*-value = 0.006), NT vs. CRC stage III (*p*-value = 0.0002), and NT vs. CRC stage IV (*p*-value = 0.0002). (**c**) Representative examples of amperograms recorded for tissue extracts from healthy patients and patients with early-stage or advanced CRC. ** = *p* < 0.01; *** = *p* < 0.001; **** = *p* < 0.0001.

As shown in Figure 6 and summarized in Table 4, quantification of MIF in protein extracts from tissue samples showed a positive correlation between MIF expression levels and disease progression. This finding is consistent with larger MIF protein expressions associated with CRC advancement, aligning with previous reports for colonic adenocarcinoma epithelium compared to normal colonic tissue [5,8,20,62].

These results prove the ability of the developed immunoplatform to efficiently differentiate between healthy tissues in early (I–II), and advanced (III–IV) CRC stages. Although it is possible to discriminate between early (I–II), and III and IV stages, there was not a clear difference between stages I and II, in line with that previously reported [20]. Since MIF is a protein highly associated with tumor cell proliferation and progression, this can justify the difficulty of distinguishing between early stages I–II. On the other hand, the examination of the quantification values obtained for matched tissues at each stage demonstrates that the immunoplatform effectively distinguishes between T and NT tissues of each stage, as well as between different stages overall (see concentrations and T/NT ratios in Table 4). If we scrutinize the tissue-matched results, it is evident that patient 1 and, particularly, patient 2 (CRC stage I) exhibited abnormally elevated MIF levels in their NT tissues compared to those from patients with more advanced CRC stages (II, III, and IV) (see Table 4). These findings can be attributed to individual heterogeneity between different tumors and CRC patients, or to incomplete resections of tumor tissue in these specific cases.

Nevertheless, despite the variability observed in the small cohort of patients analyzed, the results consistently demonstrated in all cases increased MIF expression in T tissues compared to their corresponding NT tissues, as well as higher T/NT ratio with the disease progression (see results in Table 5), highlighting the potential of the developed immunoplatform for the determination of MIF, not only to discriminate healthy from tumor tissues for patients screening, but also to distinguish and identify the stage of the disease.

In addition, these results are consistent with previous reports demonstrating that MIF is overexpressed in various solid tumors, including CRC [5,20], lung [63], breast [64,65], cervical [66], prostate [67,68], gastric [69], or head and neck [18].

Furthermore, to establish the statistical significance of the differences found between both sample groups (T and NT) and the diagnostic/prognostic potential of the developed immunoplatform, the obtained results were further analyzed using the non-parametric Mann-Whitney U test (MWU) and receiver operating characteristic (ROC) curves, respectively. According to the MWU analysis, the differences observed between the two groups were confirmed to be statistically significant in all cases (*p* < 0.05, α = 0.05).

**Figure 7:**
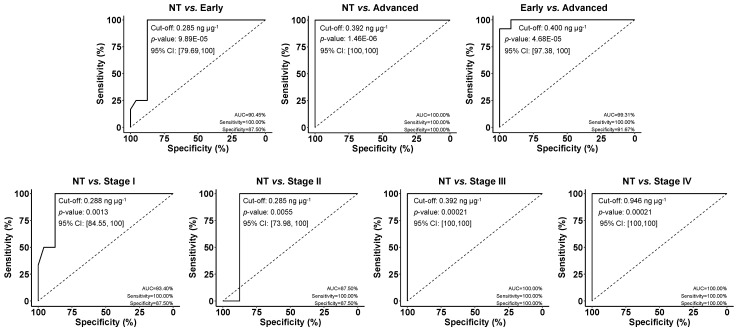
ROC curves to test the diagnostic/prognostic potential of MIF concentration determined with the developed immunoplatform in paired tumoral (T) and non-tumoral (NT) CRC tissue extracts. AUC, sensitivity, and specificity are shown in %. The optimal cut-off value, the Mann–Whitney U test (*p*-value), and the 95% confidence interval (95% CI). The optimal cut-off value for each indicated comparison is also depicted in the ROC curves.

The ROC curves displayed in Figure 7 showed full discrimination between NT and T paired tissues at advanced stages, with an area under the curve (AUC) of 100%, and a 100% sensitivity and specificity, at an optimal cut-off value of 0.392 ng µg^−1^. Furthermore, MIF tissue protein levels also possessed a high ability to discriminate early-stage T from paired NT tissues (AUC 90.45%, sensitivity 100%, and specificity 87.5%, at an optimal cut-off of 0.285 ng µg^−1^), as well as early-stage from advanced-stage T tissues (AUC 99.31%, sensitivity 100%, and specificity 91.67%, optimal cut-off 0.400 ng µg^−1^). Finally, as previously observed, the immunoplatform allowed distinguishing NT from T tissues at each CRC stage, with the highest discriminatory capacity observed at stages III and IV. In all cases, a MIF tissue concentration above 0.285 ng µg^−1^ represented the optimal cut-off to discriminate T tissues from paired NT tissues.

Despite the limited size of the patient cohort, the obtained results demonstrate the potential of the developed immunoplatform to provide clinically relevant information and the first MIF contents in tissues from healthy and CRC patients, paving the way for the next step to translate this methodology to the analysis of other minimally invasive samples, such as serum or plasma, or even exhaled breath condensates moving towards more accessible and patient-friendly diagnostic strategies.

## 4. Conclusions

This work reports the development of an electrochemical immunoplatform for the determination of MIF, a biomarker traditionally linked to inflammation that has gained increasing relevance in CRC research, as it has been associated with disease progression and unfavorable prognosis. The developed immunoplatform shows the novelty of being implemented on MMPs and focusing on oncological scenarios.

Under the optimized experimental conditions, the bioplatform showed a dynamic range (0.24 to 20 ng mL^−1^), detectability (LOD of 0.07 ng mL^−1^), selectivity, and reproducibility compatible with the quantification of MIF concentrations in relevant and challenging oncological contexts. This possibility has been confirmed by analyzing paired T and NT tissue extracts from patients with CRC using a minimal amount of sample and preparation. Statistical analysis of the results obtained shows that the methodology not only enabled early detection of CRC but also contributed to patient stratification, facilitating faster and more effective therapeutic intervention and offering a compelling alternative to conventional methods. This achievement paves the way for exploring other clinically relevant samples, such as serum, leading to a less invasive alternative, which would enable not only diagnosis, but also monitoring of treatment response, considering the role of MIF as a biomarker for chemotherapy follow-up.

In addition, the platform offers great potential for integration into portable and multiplexed electrochemical systems, considering the suitability of SPCE-based architectures for miniaturized and point-of-care devices. This adaptability could further expand the methodology toward the analysis of clinically relevant matrices such as serum or even exhaled breath condensates, enabling less invasive approaches for both diagnosis and treatment monitoring. Furthermore, future developments could explore label-free or multi-analyte detection strategies, broadening the applicability of the system to comprehensive biomarker profiling.

All these features, together with the versatility and compatibility of electrochemical biotechnologies with multiplexed approaches and miniaturized devices, position the developed methodology as a promising tool for point-of-need testing and personalized health management.

## Figures and Tables

**Table 1:** Experimental protocols evaluated for the construction of the immunoplatform for the amperometric determination of MIF.

Protocol	Steps ^1^	Total Assay Time, min ^2^
1	i: MIF + bDAb + Strep-HRP	30
2A	i: MIF; ii: bDAb + Strep-HRP	60
2B	i: MIF + bDAb; ii: Strep-HRP	60
3	i: MIF; ii: bDAb; iii: Strep-HRP	90

^1^ All steps: 30 min. ^2^ Starting from CAb-MMPs blocked with ET.

**Table 2:** Experimental variables optimized and selected values to construct the immunoplatform for the amperometric determination of MIF.

Variable	Evaluated Range	Selected Value
[CAb], µg mL^−1^	0.0–100.0	25.0
CAb_i-t_ ^1^, min	5–60	30
Protocol	1–3	3
MIF_i-t_ ^1^, min	5–150	60
[bDAb], µg mL^−1^	0.0–2.5	1.0
bDAb_i-t_ ^1^, min	5–60	30
Strep-HRP, dil	0–1/250	1/1000
Strep-HRP_i-t_ ^1^, min	5–60	15

^1^ i-t: incubation time.

**Table 3:** Main characteristics of technologies reported for the determination of MIF.

Detection Strategy	Technique	Lineal Range (ng mL^−1^)/ LOD (ng mL^−1^)	Assay Preparation Time	Storage Stability, Days	Sample	Ref.
Au electrode modified with AuNPs/TiO_2_ NPs/thionine/IgM monoclonal antibodies	Differential pulse voltammetry	0.03–230/ 0.02	12 h 10 min	30	Serum samples of rheumatoid arthritis patients	[33]
HEMT	Change in drain–source current upon analyte binding due to surface charge variation	0.018–180/ 0.018	2 h + chip preparation	--	Allergic rhinitis samples	[34]
Dopamine-coated Ab-Au (core)-Ag (shell)-SERS sensor	SERS	1–5000/ 0.09	6 h 30 min	7	Platelet-rich plasma of osteoarthritis	[45]
Polydopamine-immunocapture substrate and polydopamine-encapsulated Ab-Ag(shell)/Au(core) multilayer SERS tags	SERS	5.44 × 10^2^–2.72 × 10^4^ particles mL^−1^/ 1 exosome in 2 mL of sample solution (~9 × 10^−19^ mol L^−1^)	9 h	182	Pancreatic cancer serum samples	[46]
Sandwich immunoassay based on magnetic microparticles	Amperometry	0.24–20/ 0.07	1 h 45 min	38	Tissue extracts of CRC patients	This work

HEMT: High Electron Mobility Transistor; SERS: Surface-Enhanced Raman Scattering.

**Table 4:** Slope and t_exp_/t_tab_ values of the calibration plots constructed with the developed immunoplatform for the determination of MIF in both buffer solution and representative 100 ng of T/NT tissues extracts.

		Slope, nA mL ng^−1^	t_exp_ ^1^	t_tab_ ^1^
Buffer		182 ± 4	--	--
Tissue extract	NT (IV)	206 ± 41	1.8	2.8
	T (IV)	190 ± 20	0.9	2.8

^1^ [61].

**Table 5:** Results obtained with the developed immunoplatform for the determination of MIF in 100 ng of paired NT and T tissue extracts from patients diagnosed with CRC at different stages (I–IV).

		NT ^1^		T ^1^		T/NT Ratio		
Patient	CRC Stage	^2^ [MIF], ng µg^−1^	RSD_(n=3)_, %	^2^ [MIF], ng µg^−1^	RSD_(n=3)_, %	Patient	CRC Stage	Early_I–II_/Advanced_III–IV_ Stages vs. NT
1	I	0.24 ± 0.07	11.1	0.32 ± 0.03	4.0	1.3	1.2	1.6
2		0.377 ± 0.001	0.1	0.39 ± 0.04	4.0	1.0		
3	II	0.14 ± 0.01	3.5	0.34 ± 0.07	8.0	2.4	2.2	
4		0.17 ± 0.04	8.6	0.32 ± 0.05	6.8	1.9		
5	III	0.21 ± 0.03	5.4	0.6 ± 0.2	12.5	2.9	2.5	3.4
6		0.21 ± 0.02	4.1	0.44 ± 0.08	7.4	2.1		
7	IV	0.19 ± 0.03	6.5	1.2 ± 0.1	4.4	6.3	4.8	
8		0.21 ± 0.06	11.7	0.70 ± 0.02	1.0	3.3		

^1^ NT: non-tumoral, T: tumoral. Paired NT-T tissues. ^2^ Mean value ± ts/√n; n = 3; α = 0.05.

## Data Availability

The data that support the findings of this study are available from the corresponding author upon reasonable request.
